# Lipid-dependent deposition of alpha-synuclein and Tau on neuronal Secretogranin II-positive vesicular membranes with age

**DOI:** 10.1038/s41598-018-33474-z

**Published:** 2018-10-12

**Authors:** Oeystein R. Brekk, Alyssa Moskites, Ole Isacson, Penelope J. Hallett

**Affiliations:** Neuroregeneration Institute, McLean Hospital/Harvard Medical School, Belmont, MA 02478 USA

## Abstract

This report demonstrates insoluble alpha-synuclein (aSYN)+ aggregates in human sporadic Parkinson’s disease (PD) midbrain that are linearly correlated with loss of glucocerebrosidase (GCase) activity. To identify early protein-lipid interactions that coincide with loss of lipid homeostasis, an aging study was carried out in mice with age-dependent reductions in GCase function. The analysis identified aberrant lipid-association by aSYN and hyperphosphorylated Tau (pTau) in a specific subset of neurotransmitter-containing, Secretogranin II (SgII)+ large, dense-core vesicles (LDCVs) responsible for neurotransmission of dopamine and other monoamines. The lipid vesicle-accumulation was concurrent with loss of PSD-95 suggesting synaptic destabilization. aSYN overexpression in the absence of lipid deregulation did not recapitulate the abnormal association with SgII+ vesicles. These results show lipid-dependent changes occur with age in neuronal vesicular membrane compartments that accumulate lipid-stabilized aSYN and pTau.

## Introduction

Aging is the primary risk factor for a variety of neurodegenerative diseases, including Parkinson’s Disease (PD)^1^ and Alzheimer’s Disease (AD)^2^. Pathologically, these diseases are usually diagnosed by the presence of insoluble, higher order protein aggregates including alpha-synuclein (aSYN) and Tau, which can also be found in surviving neurons in toxin-based animal models^3^, but whether these inclusions are cause or effect remains unknown. Evidence for non-protein involvement in canonical proteinopathies can be gleaned from the gene locus *gba1*, encoding the lysosomal enzyme glucocerebrosidase (GCase), which is responsible for the conversion of glucosylceramide (GlcCer) into glucose and ceramide^4^. GCase haploinsufficiency is a major risk factor for the development of both PD^5^ and Dementia with Lewy Bodies (DLB)^6^. Human GCase function is also known to decrease in substantia nigra (SN) in both aging and PD^7^ underlying the importance of lipid homeostasis in these diseases. It is possible that lipid accumulation may promote toxicity of aSYN in human derived neurons^8^. In the broader context of neurodegeneration, protein deposits of AD-associated proteins Tau and ß-amyloid (ABETA)^9,10^ and the PD-associated aSYN^11^ are found in the brains of cognitively normal aged human subjects.

aSYN localizes to synapses where it strongly binds lipid vesicles^12,13^ and facilitates vesicular fusion through direct interactions with the SNARE family of proteins^14^. Recently, mutations in the lipid-binding domains of aSYN have been shown to cause increased lipid vesicle-binding and neurotoxicity *in vitro*^15,16^. Tau binds to and stabilizes axonal microtubules modulating their polymerization dynamics thereby controlling cellular transport of both organelles and vesicles^17^. Total Tau (tTau) and hyperphosphorylated Tau (pTau) accumulates on secretory vesicles in AD with concomitant vesicular accumulation and loss of synaptic integrity^18^. In light of these cellular functions, it is striking that lipid vesicles, membrane fragments, and cytoskeletal elements are all found within Lewy bodies in post-mortem PD samples^19^, suggesting that other pathological factors linked to organelles and broader cellular dysfunction co-exist with protein aggregation.

The current study shows that the abundance of insoluble, high molecular weight (HMW) aSYN+ aggregates in human idiopathic PD-SN are linearly correlated with loss of GCase function. To identify downstream consequences of lipid deregulation *in vivo*, we previously carried out an aging study in wild-type (WT) mice^20^. That study demonstrated a progressive, age-dependent decrease in lysosomal (GBA) and nonlysosomal (GBA2) GCase activity in the brain - and accumulation of several lipid species, including GlcCer, glucosylsphingosine (GlcSph), lactosylceramide (LacCer), and GM1a^20^. In the Hallett *et al*.^20^ study, FVB WT mice were phenotypically comparable to both BDF1- and BALB/c strains. The present report investigated changes to disease-associated proteins aSYN and TAU in the absence of canonical protein aggregation in 1.5 and 24 month old mice. In the aging mouse brain, aberrant protein-lipid interactions were discovered for aSYN and TAU. Moreover, in the older mice, aSYN and pTAU appeared in a specific subset of dopamine-containing, Secretogranin II (SgII)+ vesicles.

## Results

### Higher-order aSYN aggregates in human PD coincide with loss of GCase function, and ~24 kDa lipid-stabilized aSYN is found in aged mice with similar enzymatic deficiencies

**Figure 1:**
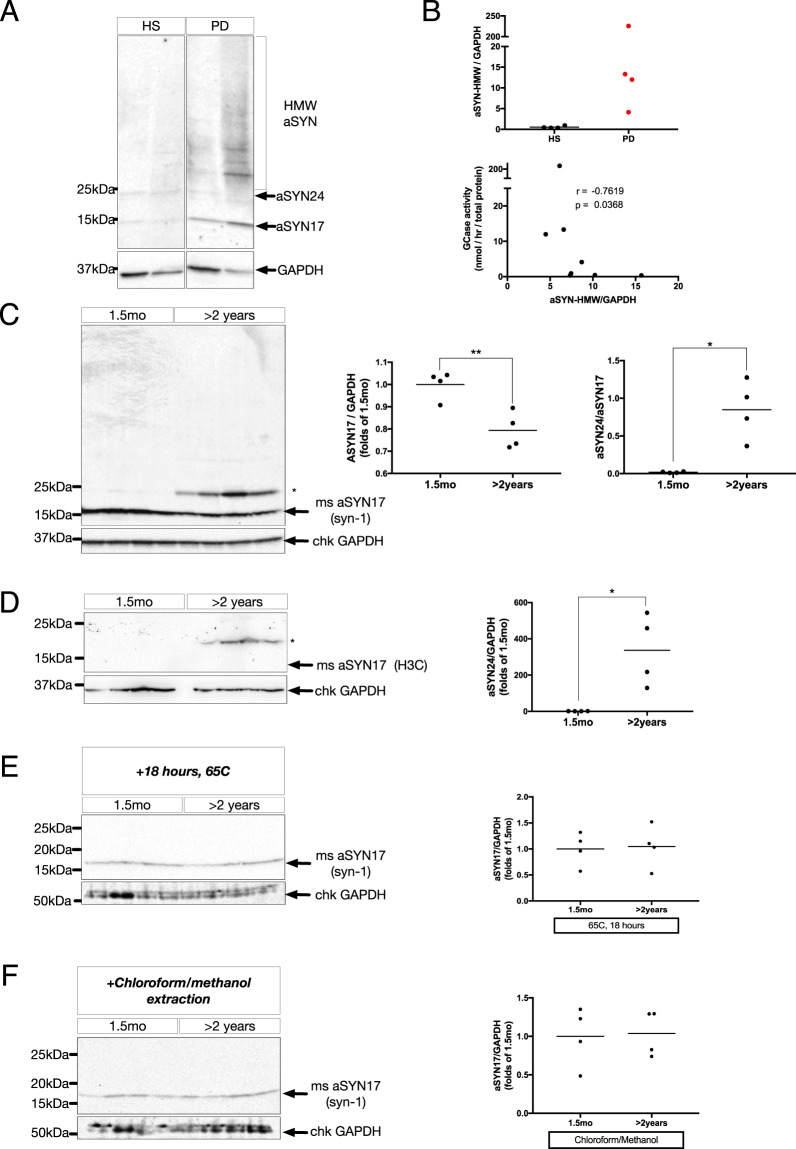
(**A**) Representative immunoblot of SDS-insoluble substantia nigra (SN) lysates from healthy control subjects (HS) and idiopathic PD-patients (PD), separated on a 4–18% Tris-HCl acrylamide gel, and probed for aSYN (syn-1). GAPDH is shown as a loading control. Lanes are cropped from a single exposure of the same gel for clarity, uncropped blot can be found in Suppl. Fig. 5. (**B**) Upper: densitometric quantifications of HMW-aSYN/GAPDH. Data represent mean with individual data points shown. Y-axis is discontinuous to better resolve variation. (N = 4/group). Lower: Cross-correlation analysis of HMW-aSYN quantifications normalized by GAPDH and glucocerebrosidase (GCase) activity. Y-axis is discontinuous to better resolve variation. (r = −0.76, p = 0.04, N = 8). (**C**) Left: Representative immunoblot of membrane-enriched, Triton-X insoluble whole-brain homogenate lysates from young (1.5 mo) and aged (>2 years) WT FVB mice, separated on a 4–18% Tris-HCl acrylamide gel, and probed for aSYN (syn-1). Asterisk denotes a higher molecular weight (~24 kDa) species of aSYN. Right, upper: densitometric quantifications of aSYN17 normalized by GAPDH, expressed as folds of 1.5 mo (**p < 0.01, n = 4/group). Right, lower: densitometric quantifications of aSYN24 normalized by aSYN17, expressed as folds of 1.5 mo (*p < 0.05, n = 4 animals/group). All data are expressed as mean with individual data points shown. (**D**) Left: Representative immunoblot of whole-brain homogenates, processed as in (**C**), probed for aSYN (H3C). Arrow indicates the expected, monomeric aSYN17, and the asterisk denotes a higher molecular weight (~24 kDa) aSYN^+^ band. Right: quantifications as in (**C**) (*p < 0.05, n = 4 animals/group). (**E**) Representative immunoblot of membrane-enriched, Triton-X insoluble whole-brain homogenate lysates from young (1.5 mo) and aged (>2 years) WT FVB mice, heated for 18 hours at 65 C to remove protein-bound lipids, and probed for aSYN using the syn-1 antibody (left). Right: quantifications as in (**C**). (**F**) Left: Representative immunoblot of membrane-enriched, Triton-X insoluble whole-brain homogenate lysates from young (1.5 mo) and aged (>2 years) WT FVB mice post lipid-removal by chloroform/methanol extraction. Right: quantifications as in (**C**). Uncropped blots can be found in Suppl. Fig. 5.

To investigate higher-order aSYN aggregates in human PD, sequential detergent extractions of post-mortem human SN were performed to isolate SDS-insoluble aSYN species, as previously described^21,22^. Utilizing a cohort of idiopathic PD patients (PD) and healthy subjects (HS) for our comparison (representative selection from previously reported cohort^7^), several high molecular weight (HMW) species were detected above the ~17 kDa monomer (aSYN17) in PD-SN that were largely absent in HS-SN (Fig. 1A,B). Comparing densitometric ratios of aSYN-HMW intensity relative to GAPDH (Fig. 1B, upper) with GCase activity measurements from the same individuals^7^, a significant, negative correlation was found (r = −0.76, p = 0.04, N = 8) (Fig. 1B, lower). To better understand the early cellular events that might precipitate higher-order aSYN aggregates in PD, young and aged WT mice were used. The 24 month old WT mice used here have significant age-associated impairments in lipid metabolism, including reduced GCase activity and elevated glycosphingolipids^20^. In WT FVB mice aged 2 years or older, there was a significant, 20 ± 5% decrease of monomeric, aSYN17 accompanied by the presence of a ~24 kDa aSYN+ immunoreactive band (aSYN24) (asterisk) significantly increased relative to young (1.5 months) littermates (Fig. 1C). Using an alternate antibody (H3C), that has been shown to preferentially label vesicle-bound aSYN in immunohistochemical assays^23^, aSYN24 was increased in aged brains, with no detectable signal in the monomeric aSYN17 range (Fig. 1D).

Upon heat-based removal of lipids from these lysates^24,25^, there was complete loss of aSYN24^+^ signal in aged animals using both Syn-1 (Fig. 1E) and H3C (Supp. Fig. 1A) antibodies. Given that robust aSYN17 immunoreactivity remained post-heating, this loss does not result from denaturing of the total protein content. Furthermore, as no age-dependent reductions of aSYN17 abundance were detected post-treatment, the monomeric pool appeared to have been increased with loss of aSYN24 (Fig. 1E, left). Chloroform/methanol-based lipid extraction^24–26^ verified the removal of aSYN24^+^ immunoreactivity (Fig. 1F, left), which similarly coincided with an enrichment of aSYN17 (Fig. 1F, right). The loss of aSYN24 was verified using the H3C antibody (Supp. Fig. 1B). In Triton-X soluble fractions, the effects of delipidation were markedly different, with multiple HMW-aSYN species being revealed post-heating, in line with previously published reports utilizing this method in soluble brain homogenates^24,25,27^ (Suppl. Fig. 1C). These soluble fractions also showed a variety of HMW-aSYN species in both young and aged animals without delipidation, but they were not different between the two groups.

Attempts at replicating our findings using a gel system of lower internal pH (~6.4 versus ~8.6) using both Syn-1 and H3C antibodies, revealed shifts in the size of age-increased aSYN HMW species detected by Syn-1, from the initially observed ~24 kDa species to a new, ~100 kDa size band (Supp. Fig. 2A). Probing with the H3C antibody, a more striking difference was observed, with a shift from ~24 kDa upwards to ~200 kDa, with significant detection of both monomeric, aSYN17, and other, intermediate species (Supp. Fig. 2B). These findings are likely due to the SDS negative charge modulating SDS binding to substrates and changing the pattern of protein migration, but differences between young and aged animals are nonetheless consistent.

### Tau is hyperphosphorylated in the aging mouse brain

**Figure 2:**
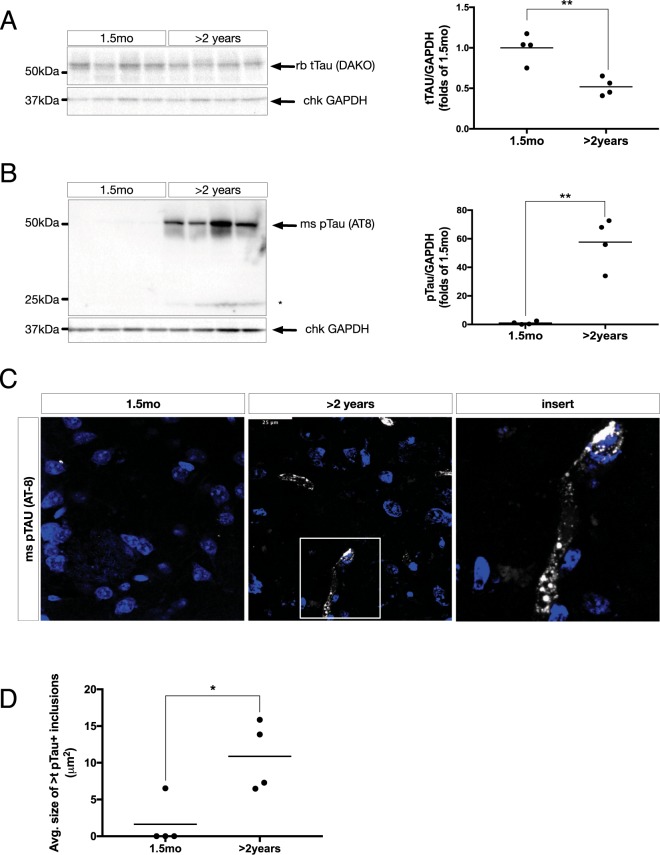
(**A**) Left: Representative immunoblot of membrane-enriched, Triton-X insoluble whole-brain homogenate lysates from young (1.5 mo) and aged (>2 years) WT FVB mice, separated on a 4–18% Tris-HCl acrylamide gel, and probed for total Tau (tTAU) (DAKO). Right: densitometric quantifications of tTAU normalized by GAPDH, expressed as folds of 1.5 mo (**p < 0.01, n = 4/group). All data are expressed as mean with individual data points shown. (**B**) Left: Representative immunoblots of whole brain homogenates, processed as in (**A**), probed for phosphorylated Tau (pTau) (AT-8). Right: quantifications as in (**A**) (**p < 0.01, n = 4/group). Uncropped blots can be found in Suppl. Fig. 6. (**C**) Representative immunofluorescent labeling of pTau (AT8) (greyscale) in coronal cryosections from young (1.5 mo) and aged (>2years) WT FVB mouse striatum (STR). TO-PRO-3 (blue) is used for nuclear counterstain. Scale bar = 25 µm. (**D**) Quantifications of average size of pTAU+ inclusions per field of view (*p < 0.05, N = 4/group).

To assess changes in protein Tau accompanying the formation of aSYN24 detailed above, lysates were probed for total Tau (tTAU) which identified a significant, 48 ± 10% decrease in relative abundance (Fig. 2A). This coincided with a >50-fold increase in Tau Ser202/Thr205 phosphorylation (pTau) (Fig. 2B). We noted the presence of a truncated, pTau fragment of ~24 kDa present, with similar relative stoichiometry to that of aSYN24 (Fig. 2B, asterisk). In the striata of aged mice, pTau+ immunovisualization revealed tangle-like structures in randomly chosen fields-of-view (Fig. 2C), and upon quantification the average size of which were significantly increased in aged mice (Fig. 2D). Upon heat-treatment of protein lysates, the pTau^+^ signal was no longer detectable (Supp. Fig. 3A). This was verified by chloroform/methanol treatment in the absence of heat-based denaturing (Supp. Fig. 3B). This effect was specific to the phosphorylated form of the protein, as chloroform/methanol-based lipid extraction did not alter the detection of tTau (data not shown).

### aSYN and pTau localizes to lipid vesicle aggregates

To investigate lipid-membrane interactions that could be relevant to the observed lipid modifications of aSYN and pTau, we immunofluorescently co-labeled said proteins with large, dense-core vesicle marker SgII in tissues of comparably aged mice of the same genotype.

**Figure 3:**
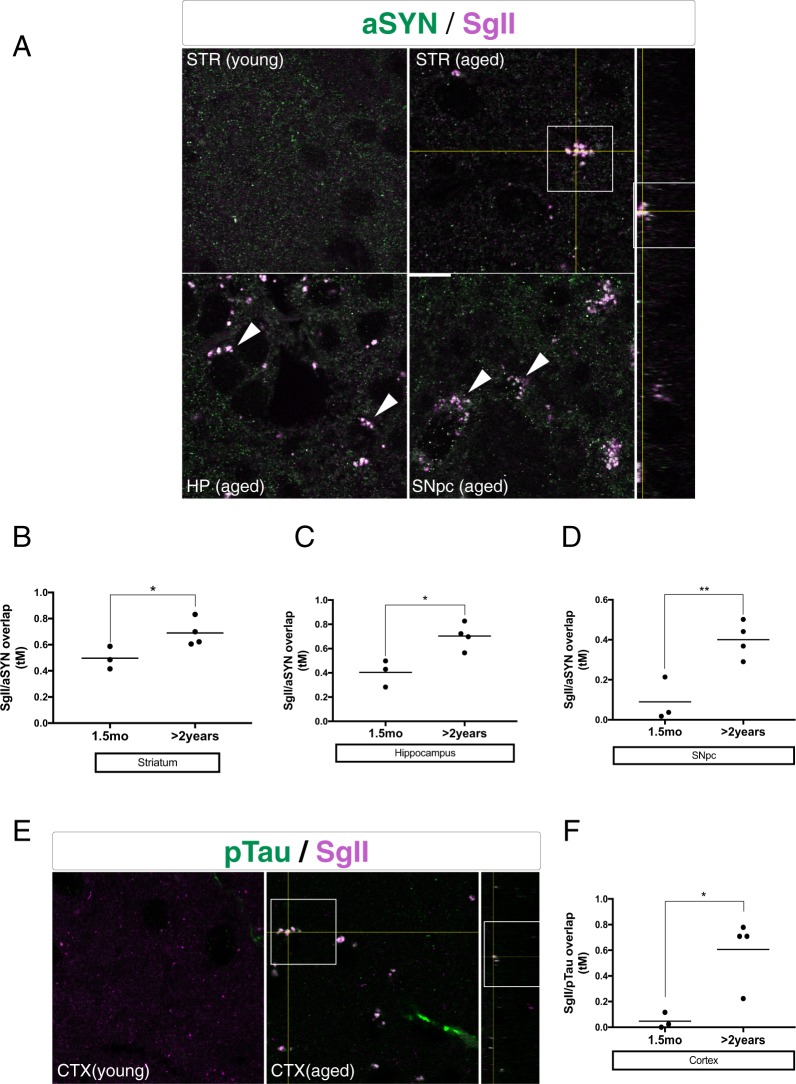
(**A**) Left: Representative immunofluorescent labeling of aSYN (H3C) (green) and Secretogranin II (SgII) (SCG2) (magenta) in coronal cryosections from young (1.5 mo) and aged (>2years) WT FVB mouse striatum (STR), hippocampus CA1 layer (HP), and the substantia nigra pars compacta (SNpc). Arrowheads indicate aSYN/SGII double-positive inclusions. Boxed insert is shown in orthogonal view on right axis. Scale bar = 5 µm. (**B**–**D**): quantifications of auto-thresholded Manders overlap coefficients of SgII in the aSYN^+^ channel (*p < 0.05, **p < 0.01, n = 4 animals/group, 15–30 images/animal). All data are expressed as mean with individual data points shown (**E**) Representative immunofluorescent labeling of pTau (AT8) (green) and Secretogranin II (SgII) (SCG2) (magenta) in coronal cryosections from young (1.5 mo) and aged (>2 years) WT FVB mouse cortices. Arrowheads indicate pTau/SGII double-positive inclusions. Boxed insert is shown in orthogonal view on right axis. Scale bar = 5 µm. **(F)** Quantifications of auto-thresholded Manders overlap coefficients of SgII in the pTau^+^ channel (*p < 0.05, n = 4 animals/group, 15–30 images/animal). All data are expressed as mean with individual data points shown.

The morphological analysis revealed age-dependent accumulation of SgII^+^ puncta in several brain regions (Fig. 3A). The SgII^+^ association with aSYN using tM overlap coefficients increased in brain regions of 24-month-old mice including the striatum (STR) (0.19 ± 0.7, p < 0.05) (Fig. 3B), hippocampus CA1 layer (HP) (0.29 ± 0.8, p < 0.05) (Fig. 3C), and the substantia nigra pars compacta (SNpc) (0.31 ± 0.7, p < 0.05) (Fig. 3D) relative to young littermates. There was a robust accumulation of pTau^+^ inclusions throughout the neocortex (CTX) of aged mice versus young, many of which colocalized to SgII^+^ puncta (Fig. 3E). The overlap-analysis in these same regions of the cortex further revealed a significant, age-dependent increase in pTau/SgII overlap relative to young littermates (0.55 ± 0.15), demonstrating that aSYN and pTau were both present at these deposits. In transgenic mice overexpressing human WT aSYN on the Thy-1 promoter (ASO mice), causing progressive alpha-synucleinopathy with formation of insoluble, HMW aSYN species^28,29^, we could detect no changes in association with SgII (Supp. Fig. 4A). Instead, we found significantly increased tM overlap of the ubiquitous vesicular marker SV2A with aSYN (0.35 ± 0.06) (Supp. Fig. 4B), in stark contrast to our findings in aged mice.

### Aberrant aSYN association is specific to LDCVs

**Figure 4:**
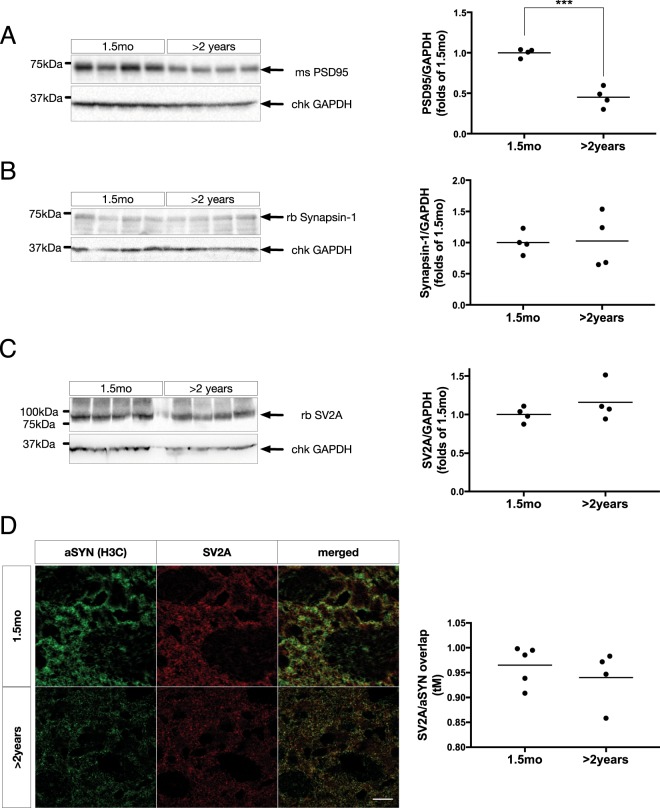
(**A**) Left: Representative immunoblot of membrane-enriched, Triton-X insoluble whole-brain homogenate lysates from young (1.5 mo) and aged (>2 years) WT FVB mice, separated on a 4–18% Tris-HCl acrylamide gel, and probed for PSD-95. Right: densitometric quantifications of PSD-95 normalized by GAPDH, expressed as folds of 1.5 mo (***p < 0.001, n = 4/group). All data are expressed as mean with individual data points shown. (**B**) Left: Representative immunoblot of whole brain homogenates, processed as in (**A**), and probed for Synapsin-1. Right: densitometric quantifications of Synapsin-1 normalized by GAPDH, expressed as folds of 1.5 mo (n = 4/group). All data are expressed as mean with individual data points shown. (**C**) Left: Representative immunoblot of whole brain homogenates, processed as in (**A**) and probed for SV2A. Right: densitometric quantifications of SV2A normalized by GAPDH, expressed as folds of 1.5 mo (n = 4/group). All data are expressed as mean with individual data points shown. (**D**) Left: Representative immunofluorescent labeling of aSYN (H3C) (green) and synaptic vesicle glycoprotein 2A (SV2A) (red) in coronal cryosections from young (1.5 mo) and aged (>2 years) WT FVB mouse striatum. Scale bar = 5 µm. Right: Quantifications of auto-thresholded Manders overlap coefficients of SgII in the SV2A^+^ channel (n = 4 animals/group, 15–30 images/animal). All data are expressed as mean with individual data points shown. Uncropped blots can be found in Suppl. Fig. 6.

Considering the accumulation of aSYN^+^/pTau^+^ LDCVs in these aged animals, we hypothesized there could be synaptic deficits related to these changes, and investigated the abundance of two pivotal synaptic proteins, PSD-95 and Synapsin-1. At the whole-brain protein level, there was a significant, 54 ± 6% reduction of PSD-95 (Fig. 4A) with no detectable change in Synapsin-1 levels (Fig. 4B). Beta-3-tubulin (TUBB3), a neuron-specific marker often used as a loading control, was significantly reduced in aged mice, to a similar extent to PSD-95 (data not shown). For this reason, all quantitative biochemical normalizations are made to GAPDH.

Total brain synaptic vesicle glycoprotein 2 A (SV2A) was unchanged (Fig. 4C), and there were no age-dependent differences in SV2A/aSYN overlap in the dorsolateral striatum (Fig. 4D).

## Discussion

Aging is the strongest driver of both sporadic and genetic neurodegenerative diseases, including PD and AD. Many protein aggregates, while undeniably linked to pathologic states of the brain, can also be part of the aging process, and other confounding factors likely exist that result in either functionally adaptive aging or pathology with evident deficits.

We have identified a lipid-stabilized aSYN species of ~24 kDa in the aging mouse brain, and a strong correlation between HMW-aSYN formation and failing GCase function in human PD. In aged mice, which present with multiple age-associated lipid aberrations, including reduced GCase activity^20^, there was significant phosphorylation of Tau protein with concurrent accumulation of lipid-modified aSYN24. As both aSYN24^+^ and pTau+ immunoreactivity could be completely removed by extraction of lipids from Triton-X insoluble lysates, this signal depends on the lipid-binding properties of these proteins in their insoluble state^24,25^. Triton-X soluble HMW-aSYN species, however, are revealed upon lipid extraction - in agreement with prior publications utilizing this method on solubilized mouse brain homogenates^25,27^. Given that the lipidated insoluble form of aSYN appeared only in the aged mouse samples, the lipidation of aSYN species may correspond to biological changes with implications for neurodegenerative diseases.

This is one of the first investigations showing effects of lipid extraction on the detection of pTau in aging or disease. Given that lipid extraction partially restored aSYN17 abundance in aged mice, it leads us to believe the ~24 kDa band reflects a lipid-modified monomer, which upon delipidation migrates with the unmodified aSYN monomer. Considering the limited migration of mouse aSYN24 observed in a lower pH gel system, it is difficult to ascertain the native size of this lipid-protein complex in the living brain. Nonetheless, the remarkable differences observed between young and old mice emphasizes the importance of aSYN in these compartments.

Both aSYN and pTau localized to a specific subset of SgII+, dopamine-containing LDCVs across a variety of brain regions, which coincided with an overall loss of PSD-95 and TUBB3. This reduction could relate to impaired synaptic trafficking and anchoring of membrane receptors and vesicles, or age-related loss of synaptic densities, which has been shown in both transgenic mouse models of disease^30–33^ and in WT animals^34^. Though we cannot rule out the contribution of cell death in the aged animals, the age-related loss of neurons in WT mice has been shown to be limited and region-specific^35^, with dopaminergic neuron loss accounting for ≤10%^36^. Whole-brain abundance of synaptic vesicle glycoprotein type 2 A (SV2A), a ubiquitous vesicular marker utilized as a readout for live PET imaging of synaptodegeneration in human patients^37^, and its overlap with aSYN, were both unchanged in the aged mouse brain suggesting that the vesicular binding of aSYN and pTau is specific to LDCVs. Considering that LDCVs preferentially contain dopamine^38,39^, such age-related cellular deficits could be involved in the pathogenesis of diseases primarily reducing dopaminergic signaling, such as PD. Conversely, as transgenic overexpression of aSYN produced strong association of aSYN to SV2A with no change in SgII overlap, the effect observed in the aging mouse brain appears to be independent of protein load. In line with this reasoning, pharmacological inhibition of GCase causing lipid abnormalities *in vivo* produces inflammatory signaling and elevated GPNMB also observed in human PD. No such GPNMB signal was evident in the pure alpha-synucleinopathy of aSYN-overexpressing transgenic mice^40^.

Taken together, these findings point to a critical role for lipid homeostasis in neuropathologies associated with aging. Interestingly, the appearance of lipid-stabilized aSYN and pTau occurred in mice without the higher-order aggregates typically observed in post-mortem pathology linked to AD, PD, and related disorders. In human WT aSYN-overexpressing ASO mice which do present with higher-order aSYN aggregates, there were no changes in the specific association with LDCVs despite the increased aSYN-binding to vesicular membranes overall. As these mice have no age-dependent changes in GCase activity^41^, this highlights the possibility that in aging, lipid membrane compositional changes trap aSYN and pTau in conditions preceding protein deposition. Indeed, brain lipid changes are associated with several neurodegenerative diseases, including PD, AD, and aging^7,42^, and aberrant vesicular membranes could constitute a lipid scaffold for aggregation-prone proteins such as aSYN and Tau. Many lines of evidence implicate vesicular transport deficits in the early pathogenesis of PD^43–46^, and certain disease-associated and artificial mutants have been shown to increase aberrant binding of monomeric aSYN to lipid vesicles through enhanced hydrophobicity of its lipid-binding domain^16^. The accumulation of SgII+ vesicles could be functionally relevant considering a recent study by Logan *et al*. detailing a crucial role for aSYN in the exocytic fusion of SgII+ vesicles^23^. In that study, a dose-dependent effect of aSYN on fusion pore kinetics was detailed with increased aSYN binding, accelerating the rate of neurotransmitter release and preventing pore closure post-release.

A view therefore emerges of altered aSYN-binding to secretory vesicles, facilitated by age-dependent changes in lipid metabolism, that could accelerate secretory vesicle binding and lead to pathology. Further support for this new perspective is provided by a recent post-mortem study describing the real content of aSYN-containing Lewy bodies in sporadic PD patients using CLEM microscopy^19^, revealing the classical Lewy bodies and neurites to contain a crowded, membranous lipid medley at their core, challenging previous views of these hallmark inclusions as purely proteinaceous. In line with this, a recent report demonstrated aSYN binding on artificial phospholipid membranes to facilitate multimerization dependent on net charge of the lipid bilayer^47^, which could be altered *in vivo* by addition of accumulating lipid species in aging and disease as has been noted in Gaucher disease cells *in vitro*^48^. The findings here in PD patients of the direct correlation between lower GCase activity and presence of HMW-aSYN, is consistent with previous work in rodents with both gain- and loss of function of *gba1*^49^, and also in idiopathic PD brain^50^. Furthermore, findings demonstrate that aSYN lipid modifications by glucosylceramide - the lipid substrate of GCase - are sufficient for inducing neurotoxicity and priming monomers for aggregation^51^. The present study supports these findings, and proposes SgII as a potential novel aSYN/pTau interactor in the aging process. Increased binding of aSYN and pTau to vesicular lipid membranes in the aging brain and in PD could result in a reduction of aSYN and Tau in other fluid compartments, and consistent with such reasoning, decreases in aSYN and pTau have consistently been reported in the CSF of patients with PD, and during aging^52–54^.

Tau has several microtubule-stabilizing roles^55^ and, in the present study, there was significant phosphorylation of Tau in aged brains that co-labeled SgII^+^ vesicles similarly to that seen for aSYN/SgII with a concurrent, overall reduction in total Tau levels. Tau phosphorylation is known to dissociate Tau from microtubules, causing mislocalization to dendritic spines, with inhibitory effects on synaptic transmission that are independent of neurodegeneration *per se*^56^ and impeded axonal transport of vesicles and organelles^57^. Tau readily binds to lipid vesicles *in vitro*^58^, and Tau interactions with phospholipid vesicular membranes are associated with both fibril formation and neurotoxicity^59^. Additionally, aberrant binding of pTau to neurosecretory vesicles and accumulation at the presynaptic terminal has been shown in several disease models^60–62^, and it was recently shown that the synaptic vesicle protein Synaptogyrin-3 is critical for this association^18^.

In summary, we have identified a significant correlation between aSYN aggregation and GCase function in human PD. In aged mice with comparable GCase deficits, we find significant Tau phosphorylation with concurrent accumulation of ~24 kDa aSYN. Both proteins localize to a specific subset of dopamine-containing secretory vesicles across a variety of mouse brain regions, which coincide with an overall loss of PSD-95. We show the states of these proteins to be dependent on lipid-protein interactions, which cannot be replicated through protein overexpression without lipid deregulation. These results highlight lipid-dependent changes occurring with age in specific vesicular membranes in mice that bind aSYN and pTau.

## Experimental Procedures

### Patients

Frozen postmortem brain tissue from male and female neurologically unaffected patients (healthy subject controls) and sporadic PD patients were provided by the Harvard Brain Tissue Resource Center (HBTRC; McLean Hospital, Belmont, MA) (cohort and diagnostic criterion detailed in Rocha *et al*., 2015a). Tissue was acquired with the full informed consent of patients or next of kin and was approved by Partners Human Research Committee/IRB. All methods were performed in accordance with the relevant guidelines and regulations.

### Glucocerebrosidase activity analysis

GCase activity measurements were obtained from previously published data sets^7^.

### Mice

All animal procedures were performed in accordance with the guidelines of the National Institute of Health and were approved by the Institutional Animal Care and Use Committee (IACUC) at McLean Hospital, Harvard Medical School. Animals were housed per standard conditions, in a dark/light cycle of 12 hour, with *ad libitum* access to food and water. Male and female FVB mice at 1.5–27 months of age (The Jackson Laboratory, Bar Harbor, USA) (RRID:IMSR_JAX:001800) were used. Human wild-type alpha-synuclein overexpressing Thy1 transgenic mice have been previously described^63^, with only males having been utilized for this study, as random inactivation of the X chromosome carrying the mutation causes diminished motor deficits and significantly reduced aSYN expression in female animals^28,64^.

Mice were terminally anesthetized by intraperitoneal injection of sodium pentobarbital (130 mg/kg) and intracardially perfused with heparinized saline. Brains were rapidly removed, homogenized in water using a handheld Polytron® homogenizer and aliquoted before being snap frozen and stored at −80 °C.

### Western blotting

Lysis of whole-brain tissues was carried out for 30 min on ice in lysis buffer (150 mM NaCl, 50 mM Tris pH 7.6, 1% TritonTM X-100, 2 mM EDTA) with protease/phosphatase inhibitors added (Halt Protease & Phosphatase Inhibitor Cocktail (100X), Thermo Fisher Scientific 1861284), and the lysates sonicated for 30 seconds (5 second pulses, on ice). Membrane-enriched fractions were pelleted in 1% Triton-X by ultracentrifugation (100,000 × G, 1 hour, 4 C), and reconstituted in lysis buffer supplemented 2% SDS. Equal amounts of protein from each fraction was separated using polyacrylamide gel electrophoresis (4–20% Criterion™ Tris-HCl Protein Gel, Bio-Rad, 3450032; or NuPAGE™ 4–12% Bis-Tris Midi Protein Gels, Thermo Fisher Scientific, WG1403A). Primary antibodies included antibodies to aSYN (syn-1, 1:1,000, BD Biosciences, 610787 (RRID:AB_398107); H3C, 2 µg/mL, Development Studies Hybridoma Bank) (RRID:AB_2618046), total Tau (1:5,000, DAKO, A0024) (RRID:AB_10013724), phospho-Tau (AT-8, 1:2,000, Thermo Fisher Scientific, MN1020) (RRID:AB_1288949), GAPDH (1:2,000; Milipore Sigma, AB2302) (RRID:AB_10615768), TUBB3 (1:2,000, Covance, MRB435P) (RRID:AB_291636), PSD-95 (1:1,000, BD Biosciences, 610495) (RRID:AB_397861), Synapsin-1 (1:1,000, Chemicon, AB1543P) (RRID:AB_212517). For delipidation, lysates were heated on a heating block (65 C) for 16 hours prior to addition of Laemmli-buffer, as previously described^25^.

Secondary, HRP-conjugated antibodies were anti-mouse (Trueblot Ultra) (Rockland, 18-8817-33) anti-chicken (Affinipure) (Jackson Immunoresearch Laboratories, 103-035-155), and anti-rabbit (Thermo Fisher Scientific, A-21206). Chemiluminescent development was done using Westernbright Sirius (Advansta, K-12043-D20), and imaging was done on a Gel Doc XR+ system (Bio-Rad, 1708195). To ensure linearity of signal, only images without saturated pixels were selected for quantifications using the densitometric western blot plug-in in ImageJ.

### Lipid removal by chloroform/methanol extraction

Membrane-enriched, Triton-X insoluble fractions were stripped of protein-bound lipids by chloroform/methanol extraction, as originally described^26^. Briefly, 40 µg total protein (making up 0.2 vol) of the interaction was mixed with 2 volumes chloroform and 1 volume methanol, vortexed for 30 seconds, and centrifuged for 5 minutes at 10,000x G (RT). The resulting phase between the upper (aqueous) and lower (organic) phases contained all delipidated proteins, and was lyophilized and resuspended in 2X Laemmli buffer.

### Immunohistochemistry

Animals were perfused intracardially through the ascending aorta with physiological saline under pentobarbital anaesthesia, followed by ice- cold 4% paraformaldehyde. The brains were post-fixed overnight in the same preparation of paraformaldehyde and subsequently transferred to 20% sucrose until sectioning. The brains were sectioned through the coronal plane at 40 µm increments, and every section throughout the striatum, hippocampus, and the ventral midbrain were collected. Immunohistochemical staining was carried out free-floating. Primary antibodies included antibodies to aSYN (H3C, 0.2 µg/mL, Development Studies Hybridoma Bank) (RRID:AB_2618046), phospho-Tau (AT-8, 1:1,000, Thermo Fisher Scientific, MN1020) (RRID:AB_1288949), SgII (1:500, Biomatik, CAU22381), and SV2A (1:500, Sigma-Aldrich, HPA007863) (RRID:AB_1857679). Secondary antibodies (goat anti-mouse and anti-rabbit) were from Thermo-Fisher Scientific (Alexa Fluor 488 (RRID:AB_26633275) & 568 (RRID:AB_141371). Nuclear counterstaining was done using TO-PRO-3 Iodide (Thermo Fisher Scientific, T3605).

### Colocalization analyses

Mounted sections were optically sectioned by fluorescence confocal microscopy, with a z-interval of 0.33 µm and a 100X objective set to 30% digital zoom. Two animals per condition were imaged per session, and settings were kept identical for all. Each full stack consisted of 15–30 images, and 2 stacks were collected per animal. For quantifications, images were background subtracted using a 15 micron rolling ball algorithm, and automatically thresholded using the bisection regression from the Coloc2 plugin in ImageJ. Manders overlap coefficients were calculated using the same plugin.

### Statistics

Graphpad Prism (v7.0) was used for all statistics (GraphPad Software, San Diego, California, USA). Two-tailed, parametric t-tests were utilized for single comparisons. For correlation analysis, Pearson’s r was computed. N (total independent replicates per group) and p values are indicated in figure legends. All graphs include mean with individual data points shown. *Post hoc* power analysis confirmed our n = 4/group to reliably detect differences >20% with 95% power (at p < 0.05).

## Electronic supplementary material

Supplementary Information

## Data Availability

The datasets generated during and/or analysed during the current study are available from the corresponding author on reasonable request.
