# Supplementary material for: Lipid-dependent deposition of alpha-synuclein and Tau on neuronal Secretogranin II-positive vesicular membranes with age
Source: Sci Rep. 2018 Oct 12;8:15207. doi: 10.1038/s41598-018-33474-z (PMC6185981; doi:10.1038/s41598-018-33474-z)
Supplement: Supplementary file 1 — Supplementary Information [file 41598_2018_33474_MOESM1_ESM.pdf]

Lipid-dependent deposition of alpha-synuclein and Tau on neuronal Secretogranin II-positive vesicular membranes with age

Oeystein R. Brekk[1], Alyssa Moskites[1], Ole Isacson[1], Penelope J. Hallett[1]

1. Neuroregeneration Institute, McLean Hospital / Harvard Medical School, Belmont MA, 02478, USA

Supplementary Figures

Supp. Figure 1

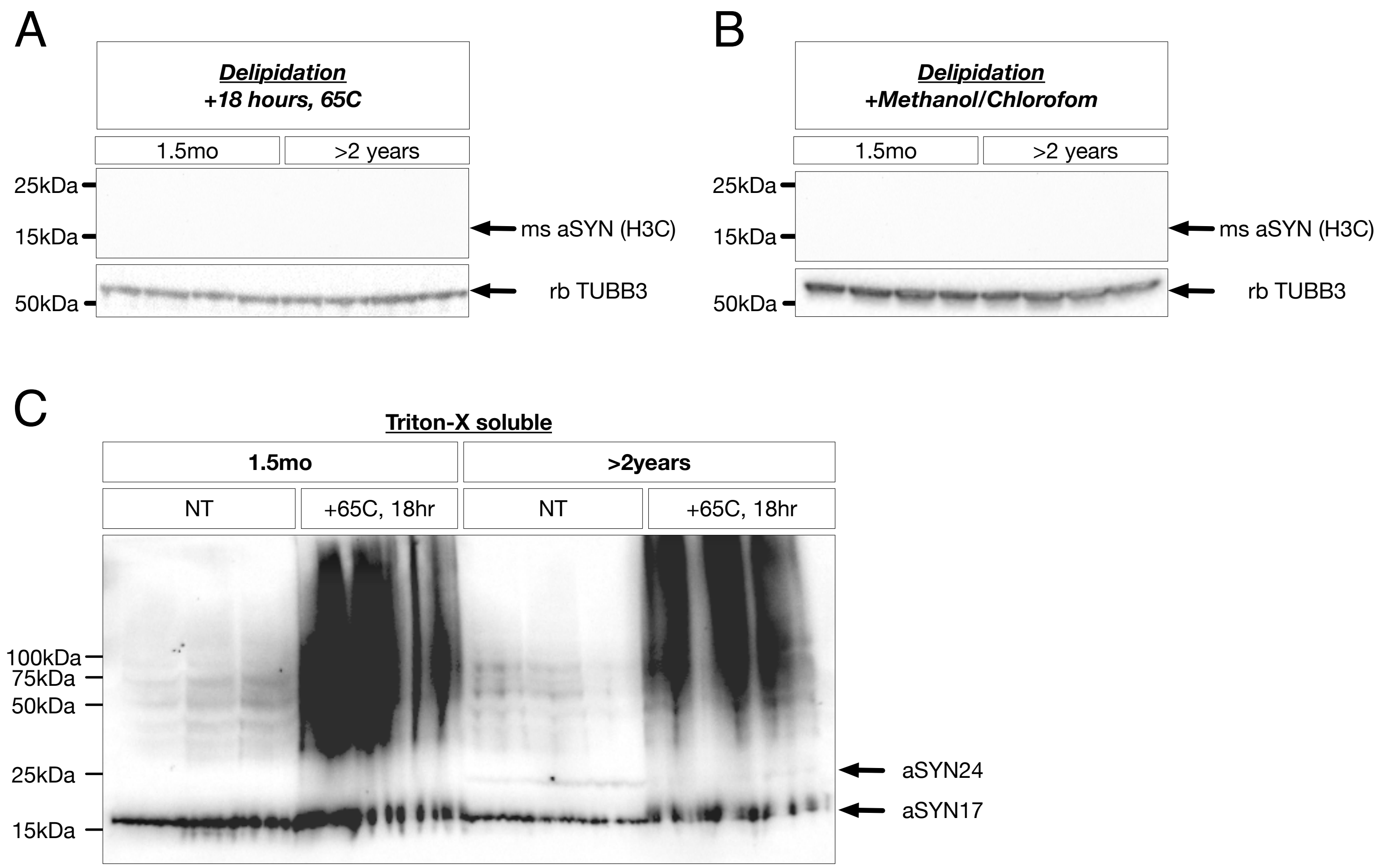

Supp. Figure 2

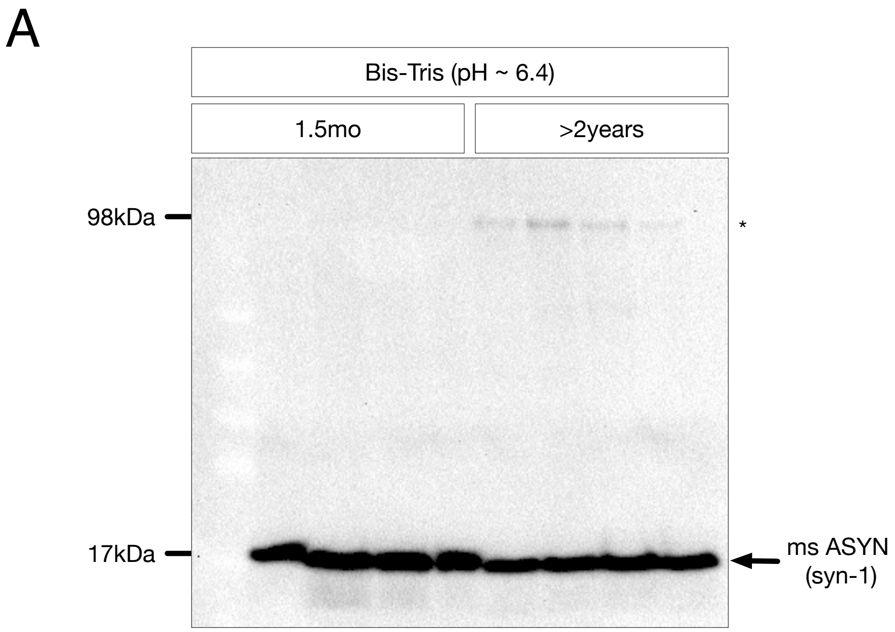

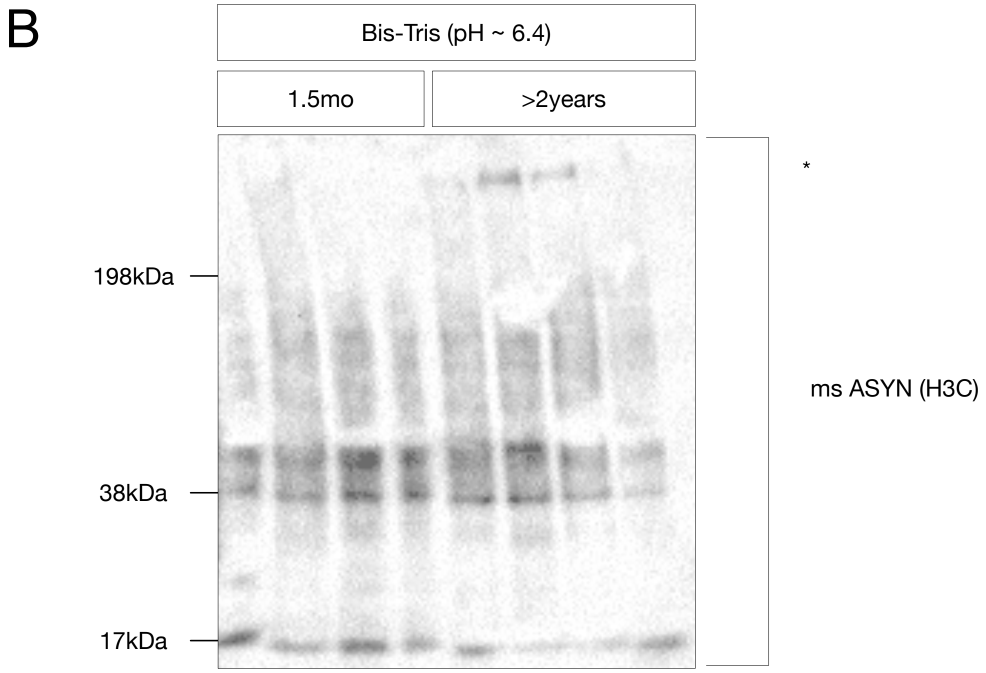

Supp. Figure 3

A

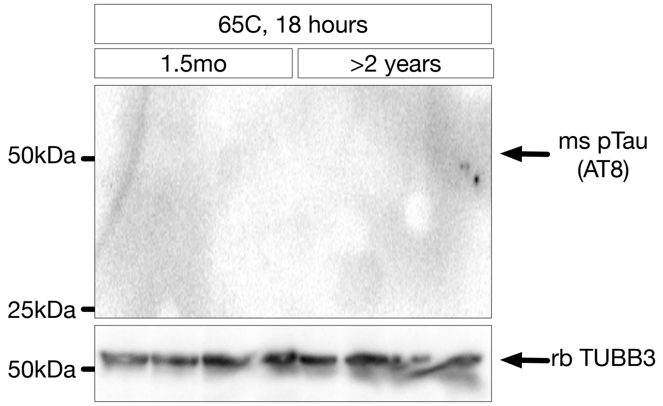

B

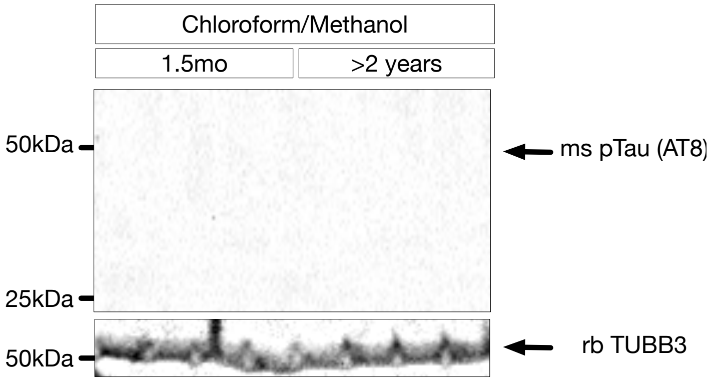

Supp. Figure 4

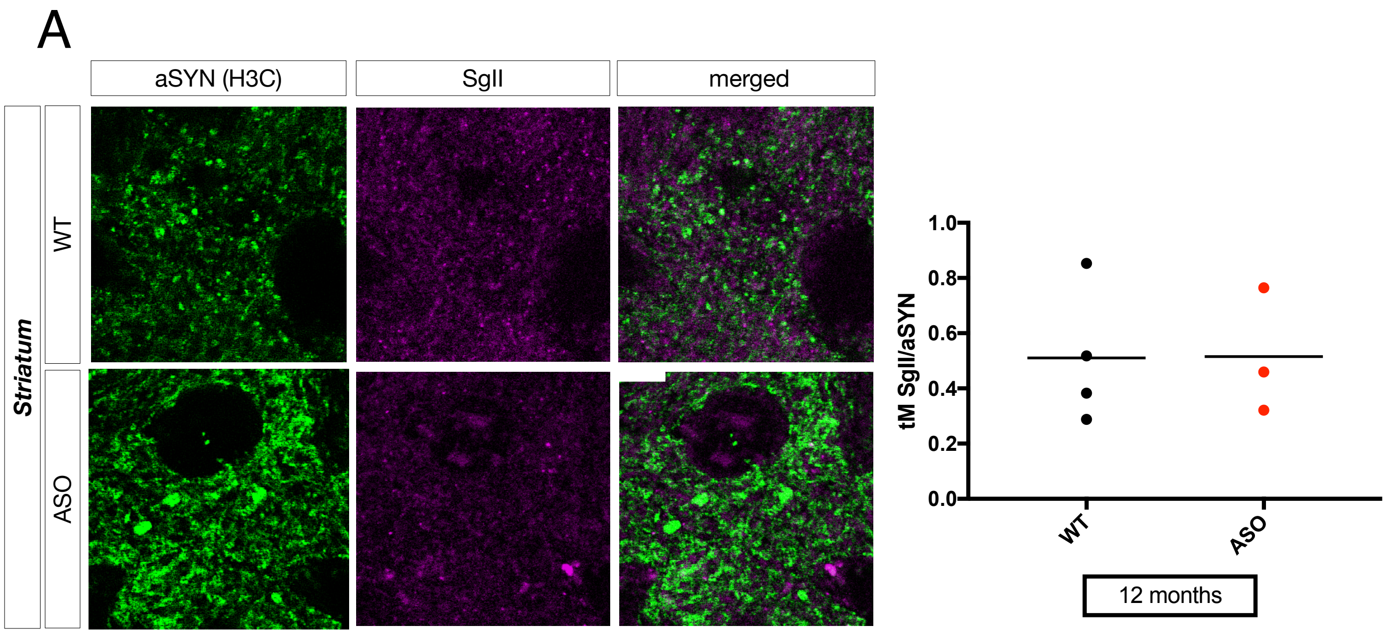

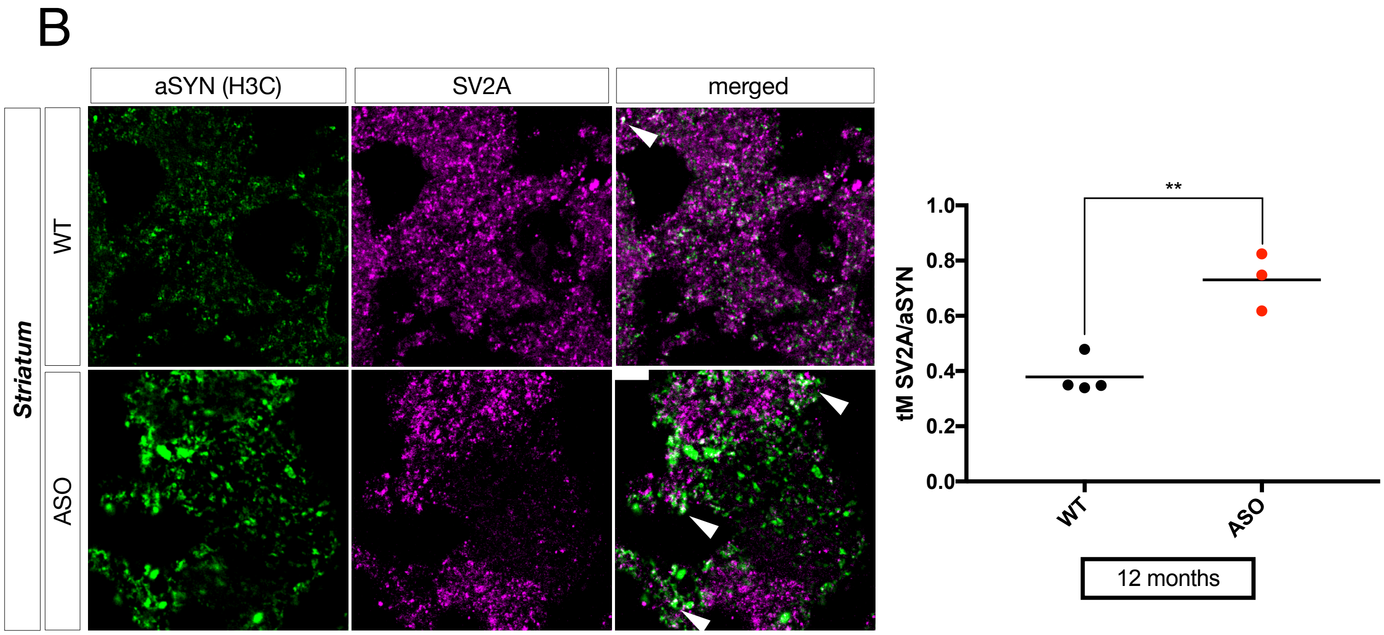

Supp. Figure 5

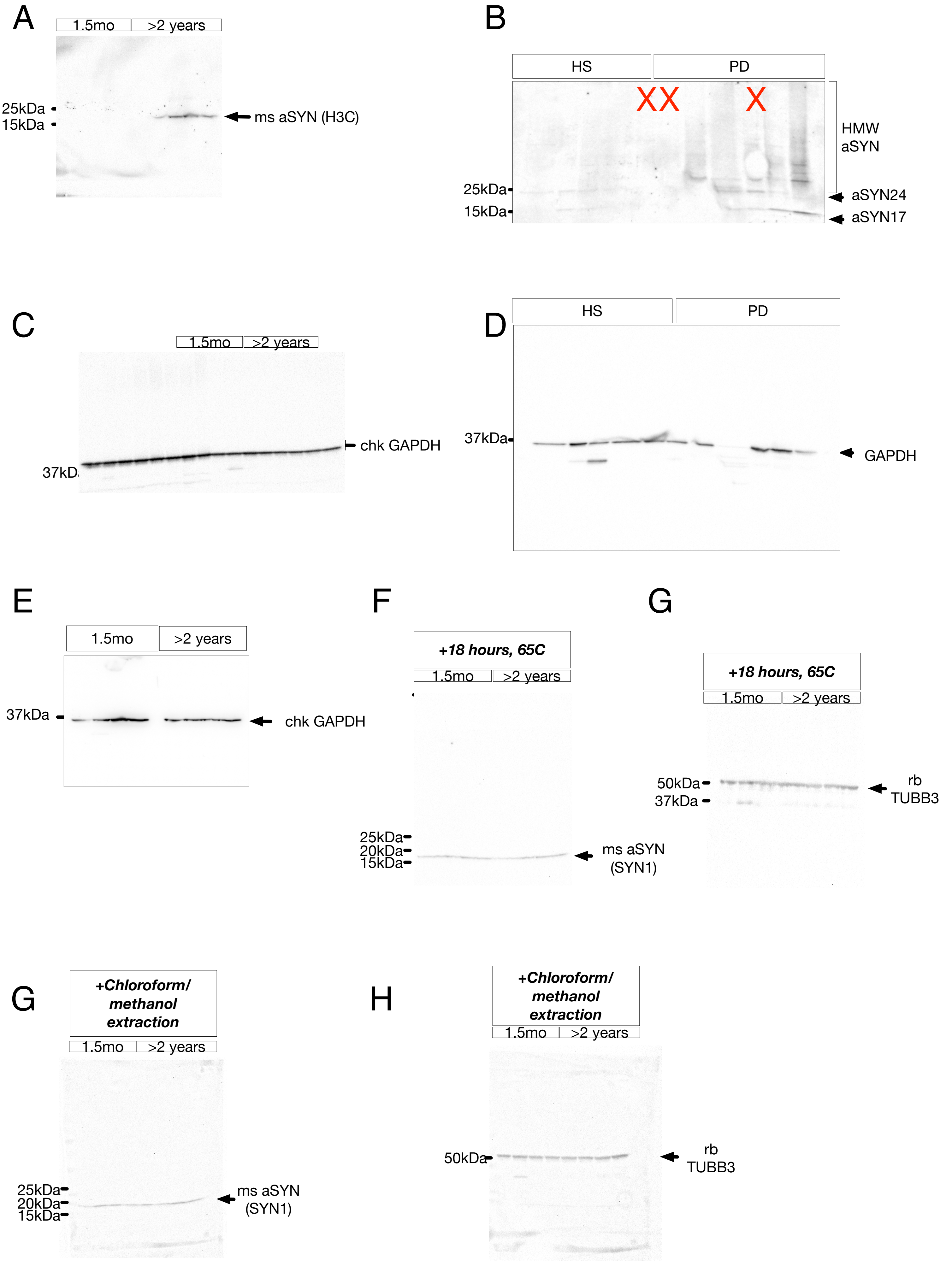

Supp. Figure 6

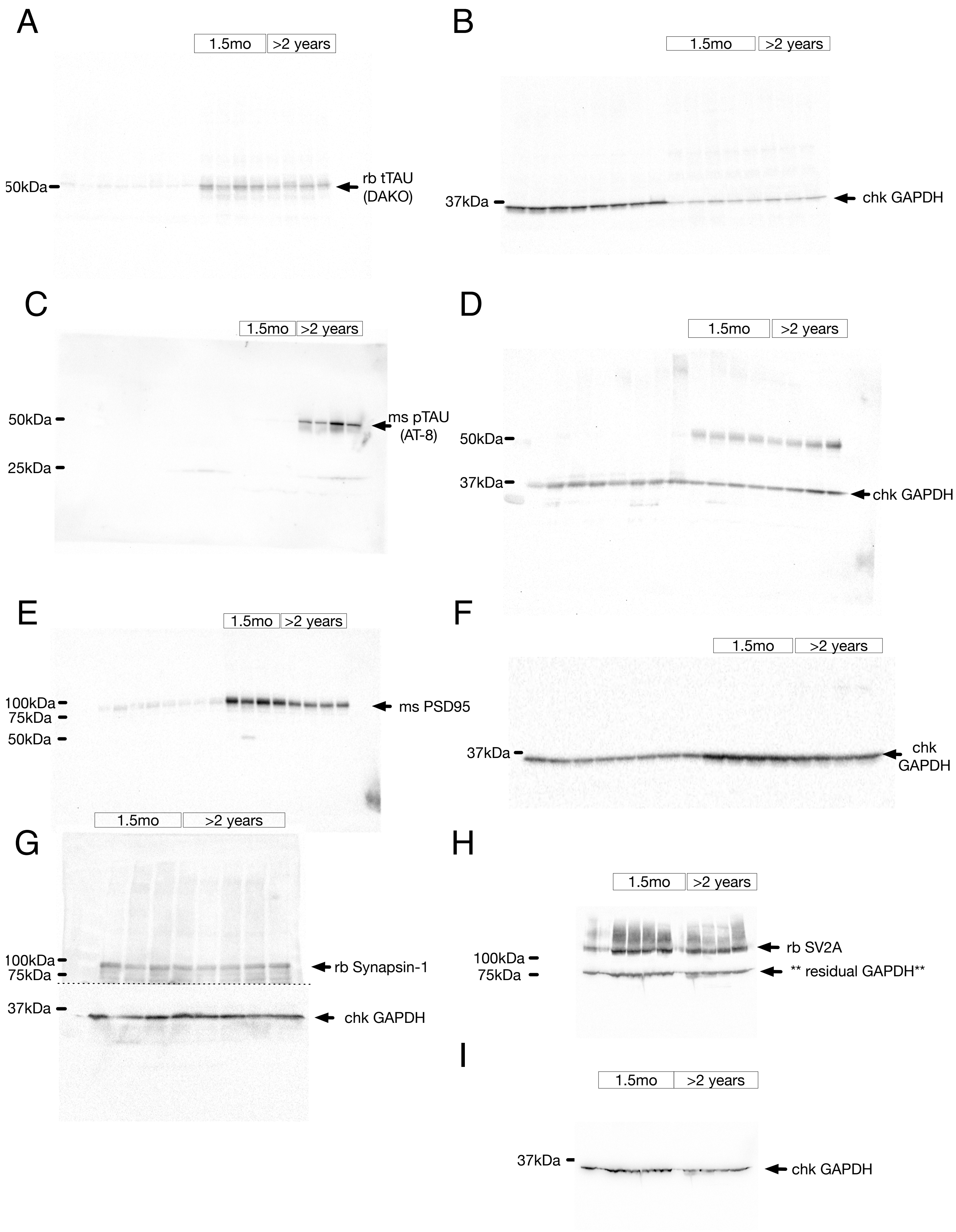

Supplementary Figure Legends

Supp. Figure 1. (A) Representative immunoblot of membrane-enriched, Triton-X insoluble whole-brain homogenate lysates from young (1.5mo) and aged (>2 years) WT FVB mice, heated for 18 hours at 65C to remove protein-bound lipids, and probed for aSYN (H3C). **(B)** Representative immunoblot of membrane-enriched, Triton-X insoluble whole-brain homogenate lysates from young (1.5mo) and aged (>2 years) WT FVB mice post lipid-removal by chloroform/methanol extraction probed for aSYN (H3C). TUBB3 is utilized as a loading control for both gels. **(C)** Representative immunoblot of Triton-X soluble whole-brain homogenate lysates from young (1.5mo) and aged (>2 years) WT FVB mice, treated as in **(A)**, and probed for aSYN (syn-1).

Supp. Figure 2. (A) Representative immunoblot of membrane-enriched, Triton-X insoluble whole-brain homogenate lysates from young (1.5mo) and aged (>2 years) WT FVB mice, separated on a 4-18% Bis-Tris acrylamide gel and probed for aSYN (syn-1), or **(B)** aSYN (H3C). Asterisk denotes a higher molecular weight species of aSYN.

Supp. Figure 3. (A) Representative immunoblot of membrane-enriched, Triton-X insoluble whole-brain homogenate lysates from young (1.5mo) and aged (>2 years) WT FVB mice, heated for 18 hours at 65C to remove protein-bound lipids, and probed for pTau. **(B)** Representative immunoblot of membrane-enriched, Triton-X insoluble whole-brain homogenate lysates from young (1.5mo) and aged (>2 years) WT FVB mice post lipid-removal by chloroform/methanol extraction probed for pTau.

Supp. Figure 4. (A) Left: Representative immunofluorescent labeling of aSYN (H3C) (green) and Secretogranin II (SgII) (SCG2) (magenta) in coronal cryosections through the dorsolateral striatum from 12-month WT and ASO mice. Scale bar = 5 μm. Right: quantifications of auto-thresholded Manders overlap coefficients of SgII in the aSYN+ channel (n=3-4 animals /group, 15-30 images / animal). Line represents mean, individual data points are plotted. **(B)** Left: Representative immunofluorescent labeling of aSYN (H3C) (green) and SV2A (magenta) in coronal cryosections through the dorsolateral striatum from 12-month WT and ASO mice. Scale bar = 5 μm. Right: quantifications as in **(A)** (**: $p<0.01$, n = 3-4 animals / group).

Supp. Figure 5. Uncropped gels from main manuscript Figure 1. Red X denotes samples that were excluded from the main analysis due to lack of aSYN-HMW signal (1 HS, 2 PD cases).

Supp. Figure 6. Uncropped gels from main manuscript Figure 2 **(A-D)** and Figure 4 **(E-I)**. In **(G)**, dashed line indicates that the membrane was cut prior to primary antibody incubation.
